# “Sex isn’t everything”: views of people with experience of psychosis on intimate relationships and implications for mental health services

**DOI:** 10.1186/s12888-021-03262-7

**Published:** 2021-06-14

**Authors:** Rebecca White, Gillian Haddock, Filippo Varese, Maria Haarmans

**Affiliations:** 1 Division of Psychology and Mental Health, School of Health Sciences, Faculty of Biology, Medicine and Health, University of Manchester, Manchester Academic Health Science Centre, University of Manchester, Manchester, M13 9PL UK; 2 Great Manchester Mental Health NHS Foundation Trust, Manchester Academic Health Science Centre, The University of Manchester, Manchester, M13 9PL UK; 3 Cathie Marsh Institute, Centre on Dynamics of Ethnicity (CoDE), Department of Sociology, Manchester Academic Health Science Centre, University of Manchester, Manchester, M13 9PL UK

**Keywords:** Psychosis, Intimate relationships, Romantic relationships, Stigma, Discrimination, Therapeutic alliance, Qualitative, Mental health services

## Abstract

**Background:**

The experience of psychosis and associated discrimination can be a barrier to forming and maintaining romantic relationships. Sexual health interventions within mental health services often focus on contraception and reducing risk. There are no known studies that seek to understand what support, if any, people who experience psychosis want regarding psychosocial aspects of intimate relationships.

**Methods:**

To address this gap in the literature, qualitative data was collected to investigate how people with experience of psychosis conceptualise romantic relationships and what support they would like in this area of their lives. Semi-structured interviews were conducted with 10 mental health service users (four women, six men) with experience of psychosis. Interviews were analysed from a critical realist social constructionism perspective using thematic analysis.

**Results:**

Stigma was a prominent theme, described as impacting numerous aspects of romantic relationships. Power imbalance within services meant participants were wary of having conversations about relationships with professionals and identified a therapeutic alliance as a prerequisite. However, abusive relationships were highlighted as a needed area for support by services.

**Conclusion:**

Services should be trauma-informed and help those in abusive relationships. The power and autonomy of people with experience of psychosis should be maintained in any discussions or interventions regarding intimate relationships. A strong therapeutic alliance is essential for any work in this area.

## Introduction

Romantic relationships are a ubiquitous part of human life and can be defined in a variety of ways. Broadly speaking, romantic relationships may be distinguished from platonic friendships due to the presence of sexual or increased physical intimacy. However, there may also be differences in terms of level of care shown to a romantic partner, romance, love and exclusivity [1]. Qualitative findings suggest people who experience psychosis associate having a partner with recovery [2, 3], however, the intimate relationship needs of this population are often unfulfilled. People with mental health difficulties experience higher rates of relationship breakdown than the general population [4]. Additionally, a meta-analysis of 1404 participants with a schizophrenia diagnosis and mean age of 39.9 years old, found just 15.6% were married [5] – considerably lower than the national average [6]. Furthermore, participants were significantly less satisfied with their sexual relationships than any other life domain.

One factor related to dissatisfaction with intimate relationships is the impact of side-effects from antipsychotic medications, such as sexual dysfunction [7]. However, prior to this, people with psychosis face numerous barriers to forming the romantic connections that lead to sexual relationships. For example, experiences of discrimination [8] and the internalisation of stigma which may make individuals feel undesirable [9]. A qualitative study which recruited people with a diagnosis of psychosis found low self-esteem and experiences such as hallucinations or becoming withdrawn were seen as barriers to both forming and maintaining connections with partners. Finally, participants discussed how sexual abuse (experienced by 36% of the 28 participants) negatively impacted self-worth and made it difficult to trust others or enjoy physical intimacy [10]. More recently another study interviewing women with ‘serious mental illness’, concluded participants expected to be rejected by those without mental health difficulties. Women minimised the importance of having a partner due to the disproportionate caretaking burden placed on them in heterosexual relationships and a desire to prioritise their own mental health. In line with previous studies, sexual trauma reduced the ability to enjoy sexual intimacy. Additionally, ‘gaslighting’ partners dismissed women’s genuine complaints within relationships as symptoms of their mental health difficulties [11]. As such, recommendations have been made for research to further investigate the intimacy needs of people with psychosis, with a view to developing trauma sensitive interventions that address stigma and enhance satisfaction in this area of people’s lives [4, 11–13].

Currently, interventions regarding the intimate relationships of people with psychosis and other mental health diagnoses tend to focus on sexual health rather than the formation and maintenance of healthy romantic relationships. There are few known psychosocial relationship interventions where sexual risk reduction is *not* the primary outcome [14–16] and no known published studies within the UK that have delivered a psychosocial intervention around romantic relationship issues to people with experience of psychosis. Additionally, and of concern, there are no known studies that have sought to understand what support, if any, people who experience psychosis want from mental health services regarding intimate relationships.

All authors of this paper view romantic relationships as a fundamental part of human life that mental health services often neglect [17]. This is something I (RW) observed whilst working in mental health services. It inspired the current research. If services are to provide support to address this area of unmet need then it is important that the delivery and content of any intervention reflects the self-defined requirements of the recipients. As such, this study aims to:

- Investigate how people who experience psychosis conceptualise romantic relationships
- Identify whether/how people with experience of psychosis would like community mental health services to provide support with romantic relationship issues

Given the nature of our research questions, our epistemological approach and wanting to gain a deep understanding of participants’ views, a qualitative methodology was adopted. Semi-structured interviews were chosen as they are ideal for understanding the views of participants and exploring sensitive topics [18].

## Methods

### Study design

Semi-structured, one-to-one interviews were conducted. We considered this study to be experiential qualitative research, as the aim of the research questions was to understand the views of people with experience of psychosis and to prioritise their voices [18]. Data were collected and analysed from a critical realist social constructionism perspective which, while acknowledging that knowledge is always mediated through social processes, suggests that discourse indirectly reflects an underlying reality. In other words, such an approach is “… ontologically realist but epistemologically relativist” [19 p. 92]. In addition, we saw our data as being co-constructed and influenced by the interaction between interviewer and participant [18].

RW is a white British, middle-class, heterosexual, 31 year old, female PhD student with a long-term partner. She has previously worked as a support worker in community and inpatient mental health services. MH identifies as a white Canadian middle-class, heterosexual, middle-aged, married woman with over 20 years of experience in the mental health field as a clinician and researcher. Her PhD in clinical psychology focused on gender and psychosis. GH and FV are qualified clinical psychologists, experienced in both conducting research, and working with people who experience psychosis clinically.

### Participants

Participants were eligible if they: 1) had either a diagnosis of psychosis or experience of psychosis that met the criteria for acceptance into early intervention services, 2) were currently receiving support from community-based mental healthcare services and 3) were aged 16 years or over. Convenience sampling was used initially, followed by purposive sampling to answer our research questions and get a deeper understanding of participants’ experiences. We considered that social identities including gender, sexuality, and ethnicity may influence people’s views and experiences and attempted to recruit a diverse sample with an even representation of men and women as well as both single and partnered participants. Participants were recruited via two community mental health services in the North West of England and through other research studies being conducted at the University of Manchester, where individuals had given consent to be contacted. Ten participants (six male, four female) were recruited. Participants’ ages ranged from 21 to 64 years (*M* = 29.7 for men, 50.0 for women). Seven were receiving support from Early Intervention Services, Recovery/Community Mental Health Teams, three were solely under the care of a psychiatrist. All had previously experienced a romantic relationship but, at the time of interview, four were in a long-term relationships. Of these, three were living with their partner. None were currently employed, three were attending educational courses. Nine participants identified as heterosexual and one as gay. Seven identified as white British/English/Irish. Two participants identified as white British with mixed heritage (Polish and Afro-American) and one as French Jewish.

### Data collection procedure

This research was carried out in accordance with the Declaration of Helsinki and ethical approval was given by Greater Manchester South Research Ethics Committee (18/NW/0755). A mutually convenient time and place was arranged to conduct the interviews. Four interviews were conducted at the participants’ home address, four on NHS sites, one at the University of Manchester and one over the telephone. All interviews were conducted by RW. A topic guide was developed by RW and MH consistent with the thematic analysis approach and wider literature on qualitative interview schedules [18, 20, 21]. Questions on the topic guide included, amongst others: ‘How do you think friendships are similar/different to the relationship you might have with a romantic partner?’, ‘Could you describe a romantic relationship that you have had, what was it like?’ and ‘Have/would you ever ask(ed) your care co-ordinator for support with a romantic relationship issue? Why/why not?’. The topic guide was revised after several interviews to include questions attentive to abuse, since this issue was raised by several participants. Most interviews were completed in one session, four were conducted over two sessions. Interviews were audio-recorded and reflective notes were written after each interview. Some participants reported initially being apprehensive about the sort of questions they might be asked but felt less anxious once the interviews were underway. The mean length of interviews was 57 min for men and 1 h 51 min for women. The lengthier interviews with women may have been due to RW being more easily able to develop rapport with female participants, being female herself and/or, becoming more skilled as an interviewer during the process of data collection.

### Data analysis procedure

Interviews were transcribed verbatim and anonymised by either RW or research interns. Braun and Clarke’s reflexive thematic analysis was adopted to identify common patterns across transcripts [18]. Transcripts were read several times before all data relating to the research questions were coded. Codes and themes were developed inductively. A hybrid approach to coding was adopted whereby some codes generated were semantic (reflecting the explicit/surface meaning of the data) and others were latent, meaning they moved beyond the literal meaning of the data to include implicit theoretical ideas such as stigma, a prominent theme in interviews [18, 22]. Once ten interviews had been coded, candidate themes were developed and data which opposed these themes were highlighted. Candidate themes were discussed with MH, then reviewed and revised by RW and discussed with the whole research team. The analysis was subject to several iterations of review and revision. When nearing completion, RW attempted to contact nine participants who had given consent for member checking. A summary of the analysis was sent to six who were contactable/agreed to take part in the process, four (two male, two female) provided feedback. The purpose was to help ensure participants’ opinions had been accurately represented. Where participants disagreed with the analysis, the goal was not to reach agreement but to try to understand the reasons for differing views [23]. Feedback was largely in agreement. Sections regarding fear of rejection by partners and the power imbalance between mental health professionals and service users particularly resonated. Some gave alternative views around parenting, caregiving in relationships, and were less critical of mental health services - these views have been incorporated into the analysis. Quotes included in the results were ‘cleaned’ by removing repeated words and non-verbal utterances to improve readability. Ellipses were used to indicate words before and/or after a quote that were not included and ellipses in square brackets were used to denote where sections of data were cut.

## Results

**Fig. 1:**
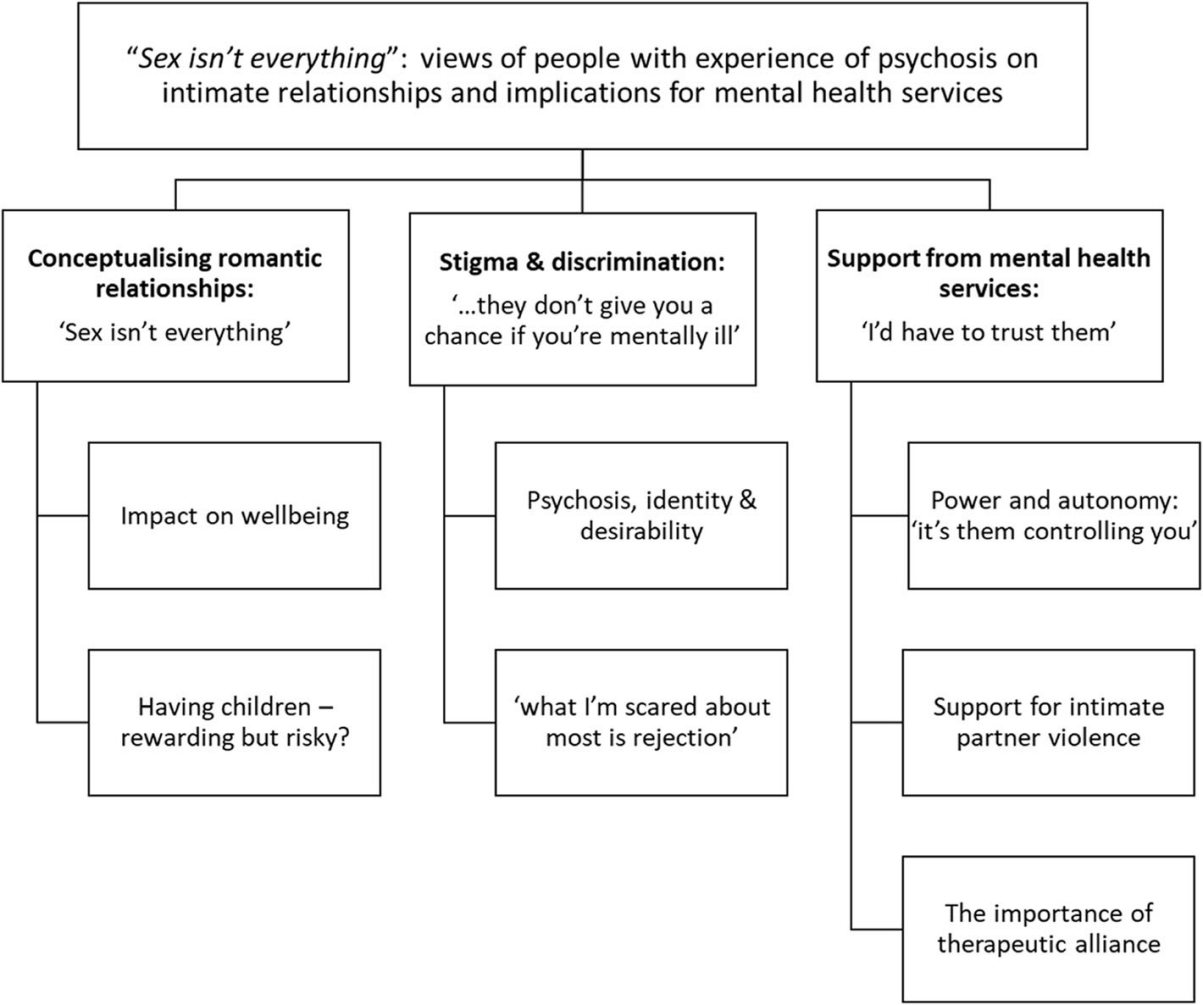
Thematic map

Three themes were developed from the data, with sub-themes as illustrated in Fig. 1.

### Conceptualising romantic relationships: ‘*Sex isn’t everything*’

Both single and partnered participants regarded romantic relationships as a fundamental aspect of human life and an aspiration for many. “ … *a relationship’s what we’re all after at the end of the day … ”* [Grant]. When asked about the difference between friendships and romantic relationships, sex was seen as the main distinguishing factor.

*It’s all very sexual when you’re in a relationship with someone* [Rhys]

*Well definitely erm intimacy (pause) I think yeah the physical side of it’s the main thing really […]* *is obviously what you don’t get with your mates …* [Lewis]

This distinction between sex and romance was also made explicit by other participants: *“… by romantic do you mean sexual? Or do y’not? Because romance isn’t really sex …*” [Anabelle], with sex seen as a physical expression of intimacy, and romance as an emotional dimension of intimate relationships. However, the importance of sex was moderated by both male and female participants’ perceptions that a deeper connection was necessary in order to have a ‘proper’, satisfying relationship.

*Well in order to be physical you gotta be mentally connected to the person as well, I believe that, where some people don’t but (laughs) but I do, there’s gotta be some connection anyway* [Mike]

*… if the main the main part of the relationship is sex, it’s not gonna go anywhere […] sex is about connecting, two people connecting, on a deeper level, very deep level …* [Jacob]

Although in member reflections it was acknowledged that some people may prefer relationships purely about sex, these were seen as unattractive by participants.

*… it’s not the be all and end all sex. Important in a relationship it’s not about sex, it’s about company, being together, talking together (pause) d’ya know what I mean, romantically together and stuff, talk about your day, your problems […] not about come home, you walk through the door ‘come on let’s have sex’* [Michaela]

One woman in particular, spoke vehemently about gender inequalities, contrasting men’s freedom regarding sexual behaviour with women’s restricted sexuality:

*… they’ve [men] got a hell of a lot more freedom t’ have a hell a’ lot more sex, an’ they talk about it […] they [men at work] talked about it non-stop […] said they go to prostitutes […] ‘yeah we go regularly blah blah’ […] y’know so they’re fuckin’ gross (laughs)[…]I’m not being used as an empty y’know fer an empty […] so you can come an fuck off, no …* [Anabelle]

This account clearly reveals gendered beliefs about men’s attitude to sexual behaviour and the need to protect herself from objectification. Whilst some men did refer retrospectively to promiscuity *“… one day I was like ‘I don’t wanna be with ya’ […] I was like sixteen or seventeen so I wanted to be out like trying out the other cuisines (laughs)”* [Rhys], none referred to this as their current preference. This may be linked to the conceptualisation of romantic relationships as much more than sex by both male and female participants, as illustrated by the quotes above. Alternatively, being interviewed by a woman may have prevented the disclosure of attitudes, such as this, if male participants felt they would be met with disapproval.

#### Impact on wellbeing

Participants were clear that romantic relationships had the potential to have both a positive and negative influence on their wellbeing, depending on the quality of the relationship *“… if you’re in a good relationship it can be a boost […] having a good laugh […] that must boost some endorphins or do you some good”* [Helen]. Partners were seen as providing emotional support: “*… with the company of somebody else it’s a bit easier sometimes to cope with life and social situations”* [Lewis]. The reciprocal nature of romantic relationships was also considered a component of wellbeing for participants. Being loved and cared for, but also caring and loving another person in return positively impacted one’s sense of belonging and connection. *“… it’s just really nice to be close to her and I can tell her what was going on with me (pause) suppose it made me feel needed, that she needed me and that I needed her in return*” [Lewis]. The importance of connection appeared especially salient for most participants given experiences of social exclusion, isolation and loneliness which are palpable in the following extracts.

*I wouldn’t have anyone if I didn’t have a relationship, I’d just be on my own in a flat* [Mike]

*It can stop people from doing stuff like taking their own lives, if some people might be that lonely they end up taking their own life, but if they got someone there it might stop ‘em* [Rich]

Participants normalised the potential of relationships to increase one’s self-worth.

*I think romantic relationships are incredibly important for anyone, regardless of whether you have a mental health problem or not I think that romantic relationships make you feel better about yourself, they give you a sense of self-worth, a sense of identity, a sense of self purpose* … [Grant]

Female participants in particular spoke about the positive impact on one’s sense of self resulting from taking care of someone in a practical sense.

*… I would prefer [ex-partner/friend] livin’ wiv me again [ …] I cooked his teas, I were doing more [ …] somebody I’m lookin’ after, that were good, it were nice feeling. But now he’s gone it’s just I can’t be arsed now [ …] when he were here I were doing it cos I had that person in the house, to look after* [Michaela]

During the member-checking process, a female participant clarified that care-taking had a positive impact only when it was felt to be reciprocal, not done under duress and where one’s own freedom was not compromised.

Where the quality of relationships was poor, they were seen as having a negative impact on self-esteem and wellbeing. *“… just noticed she didn’t really feel the same way about me as I felt the same about her [ …] it damages your confidence definitely”* [Rhys]. Additionally, some participants (both male and female) disclosed experiences of intimate partner violence which, consistent with the literature, were viewed as having a serious and long-term negative impact on trust in relationships and wellbeing [10].

*… I think cos I’ve been in bad ones […] on the whole I feel like when I’m with a fella I lose contact with my friends […] a lot of the time men just, complicate things […] I’ve been in relationships where they’ve been a bastard with me […] he was very control[ling] this guy […] an’ I just couldn’t trust ‘im […] it was awful (pause) awful, an’ since I’m really, I’ve not had another proper relationship, I think I just couldn’t trust people anymore* … [Anabelle].

Furthermore, this was the only area in which participants felt mental health services should provide support in terms of romantic relationships. This is discussed further in the theme ‘Support from mental health services’.

#### Having children – rewarding but risky?

Sex and intimate relationships were spontaneously linked by some participants to the traditional life-course milestone of having children. Accounts referred to changing priorities over time, with marriage and children becoming more important later in life. The ability to have children, create and be part of their own family unit was particularly valued by some participants.

… *up until recently relationships have been about sex, but now it’s more about family …* [Lewis]

*… marriage is something you’ve gotta work hard at, probably the hardest work but the most rewarding […] you know love, connection and many other things, having children I mean I dunno if I’ll be able, I hope to be able to have children […] it’s very important to me* [Jacob]

*… I’m glad fer what I’ve got […] I feel so blessed and lucky […] the family I’ve got now* [Catherine]

However, participants remarked that experiencing psychosis made having children and parenting more challenging due to medication side effects and vulnerability to stress. Nonetheless, where both male and female participants did have children, they prioritised them over the self and others.

*… it’s hard work sometimes cos when I want to be on my own they want to be with me. So I’ve gotta just try be there for them until they go sleep […] so I gotta like put on, I wouldn’t say like a fake smile but make it look like I’m enjoying it more that what I actually am so they feel happy themselves* [Rich]

Those without children felt they would need to be in the right *‘headspace’*, expressing concerns regarding their ability to cope with the responsibility of children and imagining they may require support with parenting from family or services.

*… I suppose I’m quite a late developer, so thinking about family came a bit later for me that other people […] you know when you’re in hospital you’re not in the right headspace to be responsible for erm (pause) on the most part anyway …* [Lewis]

Psychiatric medication was mentioned as an obstacle to conception by one participant and fears of postpartum psychosis relapse and having children taken into care was seen as a factor for consideration by another.

*… we did try for children […] it didn’t work out because she stopped with the meds and she kinda relapsed, cos the meds could of caused her problems with the baby …* [Jacob]

*… I was always a little bit concerned that I could have a puerperal psychosis relapse and then you’re in a nightmare situation of your children taken into care and stuff like that could happen, or my mother would’ve had to step in […] would I have coped with a career and children and a mental illness and a husband who may or may not have understood? I don’t know […] you potentially could harm your own children if you weren’t well, I know of a case where that happened, that would be devastating* [Helen]

Member-checking reflections reiterated these concerns and participants cited the possible hereditability of psychosis as an additional reason for choosing not to have children. Also, a need to take care of the self was seen as essential in order to fulfil a role as a parent.

Participants discussed having a family without prompting which underlines the salience of this issue. The obstacles and imagined risks associated with having children highlighted parenting as a further area where people with psychosis face stigmatisation and ‘othering’, being seen and treated as dissimilar and inferior to the general population. Implicit in the fears expressed by participants is the internalisation of dominant medical/disease model discourses and stereotypes regarding loss of control/dangerousness surrounding a diagnosis of psychosis.

### Stigma and discrimination: ‘ … they don’t give you a chance if you’re mentally ill’

Stigma was a prominent theme across the data set. Participants described stigma and discrimination relating to mental health as being pervasive, impacting not only perceptions of parenting, as discussed above, but also desirability to (prospective) partners and fears of rejection.

#### Psychosis, identity and desirability

Participants spoke about the discrimination and stigmatising views regarding mental illness they had not only encountered but come to expect. Helen recalled a time she had asked a partner what he thought of people who had experienced mental health difficulties. His response, as well as her lack of surprise, clearly reflect stereotypes associating violence and dangerousness with mental health problems:

*… I just said ‘what do you think of people who’ve had nervous breakdowns?’ […] he said ‘oh you never know when they’re gonna stab you in the back’ […] I think literally it was a fear factor […] disappointing but not really that surprising* [Helen].

Additionally, participants expressed internalised stigma and objectification related to the intersection of inferior social status/identity associated with a psychosis label as well as gendered norms regarding desirability, reflecting a sense of inadequacy and low self-worth:

*I think psychosis has planted a sort of negative voice in my head that tells me that people will always seek to use me in a relationship, that I will never get, I will never deserve happiness like my peers […] it makes you feel inadequate as a human being …* [Grant].

Several participants, both male and female made reference to lacking confidence in their desirability due to a negative body image and body shame. This was related to weight gain and linked by some to side effects from antipsychotic medication.

*… my medication causes me to eat more and I put on weight, which has knocked my confidence a bit* … [Lewis]

*… I think no one will want me ‘cause of me weight, no one will really look at me […] I were attractive then, now I put a lot of weight on […] maybe that’s why I don’t go out, I don’t know (pause) I’m ashamed how I look …* [Michaela]

While participants challenged and rejected stigmatising views regarding mental health, the discourse drawn on reflects the prevalence of such stigmatising views. For example, in the following extract, the use of the pejorative term ‘loony’ is particularly revealing regarding negative stereotypes, social identity and feelings of shame associated with a psychosis diagnosis: “… *everyone’s got mental health, it’s nothing to be ashamed about, you’re not loony …”* [Michaela]. Negative social perceptions and dominant discourses about people with mental health diagnoses shaped how participants viewed themselves in romantic relationships. Primarily, participants perceived others as viewing people with mental health difficulties as inferior and thus, undesirable partners. “… *they don’t give you a chance if you’re mentally ill …”* [Jacob].

#### ‘What I’m scared about most is rejection’

Related to prevalent misconceptions about mental health and psychosis in particular, and internalisation of illness models, participants voiced concerns about the risk of rejection. Narratives reveal how this made it difficult to disclose their diagnosis and experiences of mental distress to romantic partners due to anticipated judgement and rejection.

*I think until society changes some of its perceptions about what it’s like to be a schizophrenic, what the reality is for most of us, you know they’re not sort of raving homicidal lunatics all the time, that it’s going to be difficult because it’s against that backdrop that you drop the bombshell, so to speak, about what your diagnosis is …* [Helen]

*Well when I first got with my girlfriend I was scared about her finding out from other people […] I felt like she’d judge me, she wouldn’t want to be with someone like that. So for that reason she would’ve just left* [Rich]

As a result, participants spoke about sharing some, but not all, of their experiences with romantic partners. Other reasons male participants specifically gave for not speaking to their partners about the full extent of their mental health difficulties were not wanting to upset or worry them.

*Well in the past they’ve [voices] told me to like harm myself, to take my own life and I don’t really want to tell her that in case she judge me and like gets too worried about me and stuff* [Rich]

The views of male participants can be seen as reflecting gendered stereotypes about men being strong and protective in heterosexual relationships. In the extract below, Jacob struggles to align this gendered view of the male role with the belief mental distress would be seen by a partner as instability and weakness. Additionally, he suggests his partner may feel threatened by knowing about his experience of psychosis, reflecting stereotypes that people who experience psychosis are violent:

Jacob: … *I’ve not really spoken to my partner much about it, I’d feel kind of that she’d feel threatened if I did […] I’d prefer to keep her in the quiet about it rather than tell her*

RW: *What do you think would make her feel threatened?*

Jacob: *Just, I like to think that she sees me as a stable character and if I ruin that reputation it wouldn’t be worth it for me. I keep things as stable as possible. If she thinks I’m complete nuts, she’s less likely to be interested in me, so I kind of keep my delusions to myself, we don’t really discuss it either, but she obviously knows that, I mean she’s seen me in states where I’m not so well […] I’ve never really thought about it properly, maybe I can discuss it with her, I get the feeling that she’d prefer me to be stable healthy and well […] maybe she’d see me as weak if I did, I dunno it’s not nice to have to share that with her…*

Participants also described testing a partner’s attitude toward mental health before deciding whether to share their own experiences further.

*I do remember telling my former partner that I was struggling with, I called it ‘depression’, I said ‘I’m suffering from depression at the minute’ I said ‘I’m taking tablets I’m seeing a counsellor’ things like this erm just to try and test the waters if that makes sense to try and get some feelers for what he would how he would react to that* [Grant]

Partial disclosures or general conversations about views of people who experience mental health difficulties were a way to ‘test the water’ with partners who may hold stigmatising views and protect the self from direct judgement and/or rejection.

### Support from mental health services: *‘I’d have to trust them’*

Participants were asked what support mental health services should provide with regard to romantic relationship issues. The idea that mental health services could provide support in this area of people’s lives appeared to be an unfamiliar, fanciful concept for most and participants identified other sources of support for their relationship needs. Participant accounts seemed to reflect the nature of the medical model approach underlying service delivery where appointments are typically brief, intermittent, and focused predominantly on symptoms rather than psychosocial issues - this was a barrier to effective therapeutic relationships. Support only made sense to participants in the context of a strong therapeutic alliance, where mental health professionals worked in a collaborative way that did not compromise autonomy. When asked directly about support groups for those with marginalised experiences (e.g. abusive relationships, belonging to minority groups), participants felt these potentially did have utility.

*It could be a sort of group therapy session, cos I really think that they can be very useful […] you’re meeting people that you can empathise with more potentially …* [Grant]

*… give a group a chance to open up about their views an’ then you can discuss, I think that would be good idea* [Mike]

However, abusive relationships were highlighted as the only area where mental health services should provide support.

#### Power and autonomy: ‘it’s them controlling you’

Although member-checking highlighted aspects of mental health services that could be commended, participants perceived a power imbalance between themselves and mental health professionals. Some participants expressed strong emotions about negative treatment experiences related to a lack of appropriate support. These negative experiences damaged trust in mental health services and contributed to the view that it would be incongruous to discuss romantic relationships with mental health professionals.

*… didn’t even fuckin’ tell me that [old CPN] weren’t around […] they’d only send me a letter after I’d rang t’ fuckin’ complain that I’ve not seen somebody for so long y’know, an’ didn’t even give me replacement for ages, so they’re shit, so the last thing I’d be talkin’ t’ them about personally is relationships, when they don’t even know what I’m doin’, I could’ve been sat at home dead y’know, they wouldn’t know …* [Anabelle]

RW: *What sort of relationship would you have to have with a worker to be able to kind of talk to them about your [relationships]?*

Mike: *I’d have to trust them […] that’s why I didn’t like about hospital cos there was so many people who were new and they didn’t know me an’ they were treatin’ me like a criminal […] it didn’t help me, all they kept doin’ was punishing me, givin’ more drugs […] when you’re out in the community it’s not as bad cos you have freedom, but when you’re in a ward there’s, it’s them controlling you* …

Related to a lack of trust in services and power over treatment decisions, where participants did consider talking to mental health professionals about their romantic relationships, they were cautious about how much they shared and worried where the conversation might lead. Additionally, despite professionals’ duty of confidentiality, the official, documented nature of conversations between service user and mental health professionals, coupled with participants’ fears about what mental health services might do with the information they shared with them, were also barriers to conversations.

*… once it’s on record then it’s somethin’ that’s in black an’ white, whereby if it’s with someone ya trust y’know it won’t go any further […] everything you’ve discussed will be ya know written, dealt with to the death […] everything I’ve said and done has y’know it’s been noted down at some stage, which to me is ridiculous […] I think that’s important y’know, somebody y’can really talk to an’ if somethin’ is distressin’ ya then t’ be able t’ bring it t’ the service without fear of maybe endin’ up on in the local ward or whatever, y’know cos that’s a distinct possibility for a lot of people when they come here …* [Catherine]

*I wouldn’t mind as long as it’s not too intrusive […] I’d prefer if they didn’t ask me too much […] I have to keep my private life private […] too many people meddling in my relationship, it’s unhealthy …* [Jacob]

In order to help moderate this power imbalance and ensure people who use mental health services maintain control and autonomy over their lives, participants expressed that mental health professionals should adopt a sensitive approach when initiating conversations about relationships. Gentle enquiry, being aware people may not wish to discuss their romantic relationships and respecting this personal choice, was seen as a way to do this.

*… I think a few people might think they’re invading their privacy* [Rich]

*… be sensitive to whether the person reacts by feeling to want to open up and discuss, or wants to clam up shut down and say nothing, and respect that* [Helen]

Participants’ deployment of language such as ‘private/privacy’, ‘intrusive’, and ‘invading’ clearly illustrates the nature and extent of mistrust regarding being open about this aspect of their lives. Similarly, although participants perceived that breaking up with a partner was not easy, even when there was an awareness that the relationship was damaging or problematic, mental health professionals’ open disapproval about partners was seen as unhelpful, potentially harmful to the therapeutic relationship and a threat to the participant’s autonomy.

*… don’t think ‘e should’ve said it at all […] it’s entirely up to you, I think ‘e shoulda left me with that choice, because at the end of the day it’s my life, y’know it’s my life an’ whatever I decide to do is down to me …* [Catherine]

*… he never used to like me being with [partner] […] he thought she wasn’t good for me (pause) but I can’t live without her […] he said it, he thought she wasn’t good for me […] he gave me bad advice …* [Jacob]

Participants discussed that advice felt controlling and could lead to decisions they later regretted. Additionally, participants felt it may take time to come to the realisation that a relationship is unhealthy and that once established a romantic connection may be hard to let go of. However, this is something they wanted the power to make decisions about independently. Their accounts underline the importance of collaboration and the therapeutic alliance.

#### Support for intimate partner violence

Although generally ambivalent regarding mental health services’ role in providing support around relationship issues, one area where participants most clearly felt it was important to receive support was in cases of intimate partner violence:

… *if there’s an abuse situation going on, yes the services should step in to get help* [Helen]

*I think some people might benefit from that [support from MH services], especially if they have like a lot of domestic problems* [Rhys]

Participants supported the idea that mental health professionals should enquire about an individual’s satisfaction with their current relationships, especially if they suspected abuse. Enquiries about relationships served a dual purpose – they indicated that mental health professionals took a holistic and genuine interest in participants’ lives, which was humanising and beneficial to the therapeutic alliance, as discussed previously, but also gave opportunities for disclosures about abuse.

*I think they should ask about it to be honest, cos some people might feel trapped in their relationship, so it might be a good thing to ask erm people about it* [Rich]

*… a lot of people get raped off their partner and never open up, especially people with mental health issues […] same with beating, they get beat up […] never gets addressed, no one ever brings it up (pause) and it’s what women go through …* [Michaela]

*… support with your relationships? Only so far as if they were able to identify where it wasn’t going well and was detrimental to your health, to perhaps say: ‘Is this relationship working for you?’, to ask that kind of question* [Helen]

Additionally, participants expressed a desire to access talking therapy to process previous traumatic experiences within romantic relationships.

*I don’t talk to my workers about anything […] they don’t know anything about me past and they should do […] I don’t talk about anything about my past […] for what I’ve been through in my time it’s not an injection I need, its counselling, it’s more support, more talkin’ about getting’ me past, getting it out …* [Michaela]

*… I’ve not really got help for that properly […] they’ve got it all written down but it’s jus’ they haven’t really done anything, they give me CBT and then I wanted more CBT and then they just stopped it an’ gave me more meds …* [Mike]

Consistent with the literature [24], these accounts demonstrate the importance of mental health services adopting a trauma-informed approach and assessing for trauma. Participants here expressed a preference for talking therapy over medication to help deal with the residual and/or current impact of abuse. In addition, our findings highlight service-users’ desire for true choice regarding types of support beyond medication.

#### The importance of therapeutic alliance

Finally, participant narratives suggest conversations with mental health professionals regarding relationship issues made sense to them only in the context of a strong therapeutic alliance. A sense of knowing, trusting and having a good relationship with a mental health professional was a necessary precursor to any conversations about romantic relationship issues. This appeared particularly important given descriptions of negative experiences within mental health services in relation to the treatment/support participants had received, as described above.

*… on general if you don’t know somebody you wouldn’t really ask, that’s a personal type a thing really innit, y’know unless ya got to know someone for a while an’ ya might ask ‘em: ‘Are ya seein’ anybody?’ or whatever, it just seems like, why d’ya wanna know? (laughs)* [Anabelle]

*Close, you have to be close wiv um …* [Mike]

*… it’s to do with havin’ privacy and (pause) an’ trust […] I think with professionals I can’t always trust ‘em, because they got their own agenda …* [Catherine]

Related to the themes of power and autonomy discussed above, participants felt they were able to develop a therapeutic relationship with mental health professionals who treated them as equals and were *genuinely* interested in their welfare.

… *if they [mental health professionals] could be more sort of aware that there is a romantic aspect to a patient as well […] you’re not just a patient, you’re not just a service user, you have needs, you’re a human being, human beings need other peopl*e … [Grant]

RW: *If mental health services did offer support with romantic relationship issues, is there anything that would put you off using those services if you needed to? […]*

Lewis: *… people who’re particularly interested rather than it just being something financial for them […] so people who’re in the job because they’re passionate about it rather than being just in a job because they need to get paid*

*Bein’ more on my side, seeing my point of view […] what’s the point in telling them? I felt waste o’ space really, what’s the point in talkin’ to ‘em if they’re not gunna support me or help me really?* [Michaela]

The above extracts, particularly the phrase ‘*you’re a human being’* poignantly reflect feelings of dehumanisation and the non-collaborative nature of interactions with service providers. Helen’s account about mental health professionals’ lack of enquiry about her life below illustrates a very narrow emphasis on ‘symptoms’, neglecting other aspects of the person’s life, typical of a medical model approach to care. Both Michaela and Helen described experiences of volunteering information about a significant other, who was an important part of their lives, to be met with relative disinterest, confirming to participants that this is not an area where mental health professionals provide support:

*… they don’t tend to ask much about relationships […] I’ve tended to volunteer [relationship status] and that gets jotted down […] they are more concerned with you know display of symptoms ‘Are you paranoid?’, ‘Do you have any paranoid ideas?’, ‘How’s your mood?’, ‘How’s your sleep?’ it doesn’t tend to be in you know, top ten of the questions they ask you ‘How are your relationships?’ …* [Helen]

Michaela: … *I’m scared of him walking out an leavin’ me for good I suppose*

RW: *[…] and so if you were to kind of share that fear with one of your workers (pause) how would you feel if they kind of tried to support you with that?*

Michaela: *[…] Yeah I don’t know how they’re gunna help me on that. I don’t even know if they would help me on that, not very good my workers […] they don’t talk about [ex-partner/friend], I always talk about [ex-partner/friend] to ‘um*

RW: *Oh you do, and what do they kind of say when you bring him up?*

Michaela: *Nothing, just say ‘good’. I’ll say ‘I’m going shopping with [ex-partner/friend] after’ or ‘I’m doing this after with [ex-partner/friend] and they just say ‘good’. That’s it*

RW: *What would you like them to say?*

Michaela: *Well ‘how’s he doing?’, ‘where’s he living now?’, ‘what’s be up to?’ feedback basically […] I don’t know why they don’t do that.*

In this quote Michaela demonstrates there are aspects of her relationships that she is concerned about and could possibly benefit from support in. However, the idea of receiving support from mental health services for these concerns is perceived as unlikely due to her experience of professionals’ lack of engagement in conversations about her ex-partner. Michaela describes doing the work to initiate conversations about an important person in her life which is met with disinterest. In this instance it appears those involved in supporting her only need to engage in polite and interested conversation to begin developing an effective therapeutic relationship. However, this basic human courtesy is not offered, and so Michaela is unable to discuss and receive support for her concerns.

Time was also identified as an important facilitator for therapeutic relationships as an alliance would need time to develop and strengthen. Although some, even after knowing their mental health provider for a significant length of time, still felt more comfortable seeking support with romantic relationships from other sources.

*It depends really cos I don’t really like speaking to my care-, well I do like speaking to him but I feel like I can’t tell him everything, just because I’ve not really known him that long …* [Rich]

*… I’ve known [mental health professional] a long time so (pause) I just don’t think he’d be the right guy to do it* [Jacob]

Similarly, infrequent and short visits from mental health professionals were seen as a barrier to discussing romantic relationships.

*… that’s one of the problems with the mental health service because there’s only a certain allotted time for you to see a worker (pause) they can only deal with certain amount of problems …* [Mike]

*… when ma workers come I can’t talk like am doin’ wi’ you […] They’re just in an out five minutes ‘Heya Michaela, how are ya? Right I’ll see you next week next Tuesday’ put the diary, pick the diary up an’ they’re out. An’ that’s all a get. Where [housing officer], she sits down and talks to me properly, she can be here half an hour, forty minutes* [Michaela]

Linked to the perception that relationships were not a priority for mental health services, participants felt short visits meant there was not time to discuss relationship issues. Additionally, short visits were a perceived indicator that mental health professionals did not have a genuine interest in providing support. However, where a therapeutic alliance had been developed, a few participants described positive experiences of discussing romantic relationship issues with mental health professionals: “*I could talk to [care coordinator name] because it was at my own pace it was in my own house, I felt comfortable, I felt secure, I felt safe”* [Grant].

## Discussion

This study, the first to our knowledge, aimed to investigate how people with experience of psychosis conceptualise romantic relationships and explore if, and how, they would like to be supported by mental health services in this area of their lives. Romantic relationships were conceptualised as a fundamental aspect of life. While sex was seen as a distinguishing feature, in line with the literature on intimacy, emotional and cognitive intimacy were also viewed as a central and desirable dimensions of romantic relationships [25].

Themes of prejudice, discrimination, and rejection due to experience/diagnosis of psychosis were prominent and cross-cutting in our findings. Indeed, reductionist terms such as ‘schizophrenic’ are still common and appear alongside graphic and emotive descriptions of violence in the media, contributing to persistent beliefs within the general population that people who experience psychosis are inherently dangerous [26]. A previous study found people with a diagnosis of schizophrenia felt a need to conceal their diagnosis from friends and family [27]. The present study found this was applicable within romantic relationships also. A novel finding is how some navigated this predicament – using partial disclosures or enquiring about a partner’s attitude towards mental health tangentially to gauge reaction whilst protecting themselves from judgement and rejection. Our findings are also consistent with previous research which has linked experienced stigma with internalised stigma and low self-esteem [9]. Although some participants in the current study rejected stereotypes, medicalised language was still used to discuss mental health, suggesting an illness model of psychosis had been internalised. Belief in biological rather than social causes of psychosis has been linked to increased negative attitudes towards people with psychosis diagnoses [28, 29] and thus may have contributed to feelings of undesirability among participants. It is important mental health services are aware that people who experience psychosis may expect rejection from romantic partners due to internalised stigma, discrimination and lack of self-worth [8, 9]. Consistent with the literature, parenting was another area where the impact of stigma was apparent in participant accounts. An Australian study found many parents with a mental health diagnosis felt others believed they were incapable of being a good parent due to their mental health. Although the majority rejected this idea and reported being a parent was a motivating factor to better manage their mental health, this perceived stigma was still associated with negative self-reported parenting experiences, with mothers more likely than fathers to internalise stigma [30]. Subsequently, the authors recommended interventions to target stigma [30]. However, their finding that participants had also experienced discrimination from services, and our key sub-theme regarding power imbalance leading to mistrust of services, highlights the importance of any such interventions being wanted, and delivered in the context of a strong therapeutic alliance that empowers recipients.

Participants in this study largely rejected the idea of mental health services providing support regarding romantic relationships, however this is not to say mental health services should abandon any idea of providing support in this area of people’s lives, or that some service users would not benefit from support, particularly regarding internalised stigma and self-esteem. Rather, rejection of support was mainly due to power imbalances and a lack of an effective therapeutic alliance. Interestingly, in our previous qualitative study, mental health professionals identified age and gender as important influences on relationship dynamics [17]. However, participants in this study focused on relational aspects such as time spent during visits/appointments, and criticised professionals’ emphasis on symptomatology over their personal lives. The power imbalance between mental health professional and service user experienced by participants had also damaged trust. Trust enables the management of anxiety in times of vulnerability, but when we suspect others are not acting in our best interests trust in undermined [31]. In mental health services trust can be compromised by a lack of time for relationship-building between service user and professional as well as involuntary treatment [31]. Our previous study found mental health professionals may be concerned about the risk to service user wellbeing and their career if supporting someone with a romantic relationship issue had a negative outcome [17]. Managing these conflicting interests and developing trust is essential for a therapeutic alliance, which our findings indicate is a prerequisite for any support/work around romantic relationships. Empowering service users regarding decision making has also been linked to trust [32]. In line with the literature, participants in this study valued and wished to protect their autonomy in any discussions or decisions about romantic relationships. Mistrust and concerns they would not be given this power appeared to be the primary reason participants did not want support from mental health services in this area of their lives. A recent ethnographic study within an early intervention team concluded when managers engage in reflective leadership and resist organisational policies seen as counterproductive with the goals and values of the service, workplace trust can develop between staff i.e., supportive relationships and shared decision making where possible. This facilitated knowledge sharing and collaboration. Staff were able to take risks trying new approaches which aimed to and succeeded in, generating trust between the service and clients [33]. Just as trauma-informed services need to provide trauma-informed training and supervision to staff [34], mental health professionals need to be able to trust in their service to support them in order to facilitate trust between themselves and service users. Regarding support with romantic relationships specifically, mental health professionals need to have trust in management support and their career security to feel confident doing work in this area that is empowering and does not compromise service users’ autonomy.

The only area that participants did agree mental health services should provide support in was instances of abusive relationships. The prevalence of interpersonal abuse in people who experience psychosis and schizophrenia is known to be high [35, 36], but less is known about intimate partner violence specifically. A systematic review and meta-analysis which aimed to understand the prevalence of domestic violence in people with mental health diagnoses found only three studies measured domestic violence in people with schizophrenia/psychosis. However, these reported a prevalence ranging from 43.8–83.38%, with one finding women with psychosis were more than three times as likely to report violence from a partner in the past year compared to those without a mental health diagnosis [37]. Intimate justice theory suggests stigma, inequity and discrimination can reduce an individual’s beliefs about what they deserve and are entitled to within a relationship [38]. By extension, marginalised groups, such as those receiving a psychosis diagnosis, might be more likely to have lowered expectations of partners due in part to internalised stigma and dominant negative discourses surrounding psychosis. This is similar to our findings on expectations of rejection and warrants further research. Unfortunately, a recent meta-review concluded there are few interventions which specifically address intimate partner violence in women who have a mental health diagnosis [39]. Based on this finding, Van Deinse and colleagues called for professionals working in both mental health services and domestic violence/sexual assault services to receive training in delivering interventions that meet the intersectional needs of this group [39]. Although less researched, men also report abuse from romantic partners [40]. Male, in addition to female participants in our study, disclosed intimate partner violence, highlighting both men and women with experience of psychosis may benefit from support in this area. Some participants endorsed the idea of peer support. Peer support may be a helpful approach to reducing internalised stigma due to increasing social connectedness, providing role models with shared experience and removing the hierarchical power dynamic of mental health services [41].

### Limitations

This study recruited a relatively diverse sample regarding age and included those who were single, partnered, as well as those with and without children. However, we were not able to recruit a more representative sample of social identities such as ethnicity and sexuality. The findings present a somewhat broad view of the beliefs people with experience of psychosis have on the topic and may be transferable to similar participants receiving support from community mental health services in the UK. However, future research may benefit from recruiting more homogenous samples, to better understand any specific needs of these groups. Additionally, further attention should be paid to whether and how social identities such as age, gender, ethnicity and sexuality influence how people wish to be supported in this area of their lives.

## Conclusions

Findings from this study indicate that people with experience of psychosis view romantic relationships as a vital part of life. However, mental health services were often seen as being uninterested in the romantic relationships of participants and more interested in symptoms. This juxtaposition in attitudes contributes to the dehumanisation of mental health service users and is damaging to the therapeutic alliance. Simple ways professionals can overcome this and foster more egalitarian therapeutic relationships were highlighted, for example, by showing interest and engaging in conversation about the significant others in service users’ lives .

Stigma and discrimination regarding people with experience of psychosis were prominent in participants’ narratives and were seen as barriers to establishing romantic connections. Use of psychosocial explanatory frameworks may help overcome the pervasive stigma and discrimination around psychosis and promote more compassionate and positive attitudes [29]. Whilst it is clear many participants in this study would not currently welcome the involvement of mental health services in this area of their lives, there was agreement support should be provided to help those in abusive relationships, highlighting the importance of routine enquiry and services being trauma-informed. Future research and practice could draw from intersectional approaches to working with intimate partner violence [42] to address this identified need [41].

## Data Availability

The dataset generated and analysed during this study are not publicly available as interview transcripts contain sensitive and potentially identifying information which participants have not given consent to be shared in full.

## Abbreviations

CBT: Cognitive Behavioural Therapy
CPN: Community Psychiatric Nurse
